# Culturally Tailored Mindfulness Workshops: Promoting Brain Health Among Latino/a/e/x Caregivers

**DOI:** 10.1002/alz70858_104375

**Published:** 2025-12-26

**Authors:** Valentina B Flores Diaz, Javier Neira Salazar, Maria del Carmen Rosales, Hector Portillo, Aracely Portillo, Michelle McKoy, Maria C Mora Pinzon

**Affiliations:** ^1^ School of Medicine and Public Health, University of Wisconsin ‐ Madison, Madison, WI, USA; ^2^ University of Wisconsin ‐ Madison, School of Medicine and Public Health, Madison, WI, USA; ^3^ Department of Medicine, Division of Geriatrics, School of Medicine and Public Health, University of Wisconsin‐Madison, Madison, WI, USA; ^4^ Padres e Hijos en Accion, Madison, WI, USA; ^5^ Bridge Lake Point Waunona Center Neighborhood, Madison, WI, USA; ^6^ Wisconsin Alzheimer's Institute, University of Wisconsin ‐ Madison, Madison, WI, USA

## Abstract

**Background:**

Latino/a/e/x caregivers of children or individuals with disabilities or developmental delays face significant stress, which increases their risk for cognitive decline, including Alzheimer's disease and related dementias. While Mindfulness‐Based Interventions effectively reduce stress and promote brain health, culturally tailored programs for this population remain limited. The purpose of this study is to describe the acceptability and feasibility of culturally tailored mindfulness workshop that promotes brain health among Latino/a/e/x caregivers.

**Method:**

Two workshops were conducted in 2024consisting of six weekly one‐hour sessions incorporating mindfulness techniques. Sessions were conducted in Spanish, with supporting materials tailored for cultural relevance and practical application. In the final session of each workshop, participants shared feedback on how the program helped them better understand their stress responses and behavioral patterns.

**Result:**

A total of 23 caregivers participated in the workshops hosted by both organizations, with 69.57% attending at least five of the six sessions. During the final session, participants reported a deeper understanding of how stress manifests in their daily lives, including its physical effects such as headaches, fatigue, and muscle tension, as well as its emotional impact, such as irritability and feelings of being overwhelmed. Also, participants highlighted the value of emotional regulation techniques in diffusing tense situations with their families. Others noted that the group discussions fostered a sense of belonging, helping them feel less isolated in their caregiving challenges. Host organizations emphasized that the program was highly relevant to the caregivers' needs and aligned with their cultural values and experiences. However, both programs faced challenges related to scheduling. Sessions rarely started on time, as adjustments were often required to accommodate participants' arrival and to ensure adequate session completion. This logistical issue impacted the overall timing and required flexibility from facilitators.

**Conclusion:**

These pilot programs demonstrate that culturally adapted mindfulness workshops are feasible and effective in addressing stress among Latino/a/e/x caregivers of children with or without disabilities. These interventions show promise for improving brain health and well‐being in underserved populations.

